# Sustained Improvement of Negative Self-Schema After a Single Ketamine Infusion: An Open-Label Study

**DOI:** 10.3389/fnins.2020.00687

**Published:** 2020-07-01

**Authors:** Gregor Hasler, Samir Suker, Georgios Schoretsanitis, Yoan Mihov

**Affiliations:** ^1^Psychiatry Research Unit, University of Fribourg, Villars-sur-Glâne, Switzerland; ^2^Translational Research Center, University Hospital of Psychiatry and Psychotherapy, University of Bern, Bern, Switzerland; ^3^Psychiatry Research, The Zucker Hillside Hospital, Northwell Health, Glen Oaks, NY, United States

**Keywords:** ketamine, depression, self, BDI-II, MADRS

## Abstract

Conventional antidepressants have several important limitations, including a lack of direct effects on negative self-schema, which is at the core of Beck’s cognitive theory of depression. Based on previous studies showing a positive effect of ketamine on negative cognition, we compared reductions in negative self-schema between responders and non-responders to a single infusion of ketamine. In an open-label study, 26 participants with treatment-resistant depression received 0.5 mg/kg ketamine via infusion. Depression symptoms were assessed at baseline, 24 h, and 7 days after treatment with Montgomery-Åsberg Depression Rating Scale (MADRS) and Beck Depression Inventory (BDI-II). Nine of the 26 participants fulfilled response criteria after 24 h. Of these, eight still fulfilled response criteria after 7 days. Response was defined as a reduction in MADRS total score of 50% or more. Responders improved significantly more than non-responders both 24 h and 7 days after ketamine treatment on the following BDI-II items: item 1 (“Sadness”), item 7 (“Self-Dislike”), and item 8 (“Self-Criticalness”). These results suggest an important therapeutic effect of ketamine on negative self-schema, which is a fundamental cognitive aspect of depression. This effect is unique and might be associated with ketamine’s profound effects on neuroplasticity. Small sample size and lack of a placebo control group are the major limitations of this study.

## Introduction

Conventional antidepressants are thought to produce their impact on clinical symptoms by increasing monoamine concentration in the synaptic cleft. These drugs continue to be the first-line choice in the pharmacotherapy of depression. However, they have major shortcomings, such as a delayed onset of action and a high rate of non-response.

The discovery of ketamine as a rapid antidepressant has caused a paradigm shift in depression research and treatment (Krystal et al., 2019). A single dose of ketamine was shown to produce rapid, profound and surprisingly durable antidepressant effects (Berman et al., 2000). Importantly, studies demonstrated that ketamine was effective in antidepressant non-responders (Zarate et al., 2006). Aggregate analysis of placebo-controlled trials suggested that ketamine has a specific profile of action (Ballard et al., 2018). Overall, ketamine improved depressed mood, negative cognition, anhedonia, and suicidal thoughts better than tension, reduced appetite, and impaired sleep.

In this study, we used data from uncontrolled, naturalistic treatment of severely depressed patients with a single infusion of ketamine. We used the Montgomery-Åsberg Depression Rating Scale (MADRS) to evaluate response. Participants were categorized as responders if they displayed an improvement of 50% or more of their baseline MADRS score after 24 h, and as non-responders otherwise. In a next step, we analyzed the profile of symptom improvement after ketamine treatment. To this end, we examined differences in symptom improvements between responders and non-responders in Beck Depression Inventory (BDI-II) 24 h and 7 days after treatment.

## Methods

### Study Sample

All participants provided informed consent prior to the ketamine treatment. The retrospective exploratory analysis of clinically assessed data was approved by the local ethics committee Bern (Kantonale Ethikkommission Bern). The study was carried out in accordance with the guidelines of the Swiss Academy of Medical Sciences (SAMW) and applicable Swiss law.

We included the entire cohort of 26 persons that received ketamine for treatment-resistant depression in a tertiary treatment center of the University Hospital of Psychiatry and Psychotherapy, University of Bern, Switzerland. Inclusion criteria comprised a diagnosis of a severe mood disorder following ICD-10 criteria, treatment resistance (non-response to at least three different treatments), and age greater than 18 years. We excluded participants fulfilling the following criteria: psychotic symptoms or full manic episodes in the past, misuse of ketamine or other illegal substances, ECG abnormalities (higher grade blockages and pre-existing QT/QTc extensions), any heart disease, acutely increased risk of suicide 14 days before treatment, any acute somatic disease, history of severe somatic disease, history of epileptic seizures, known ketamine hypersensitivity, pregnancy, thyroid gland disorders, severe cognitive deficits, neuropathic pain, glaucoma, anticoagulants, any coagulation disorder, and any contraindications against intravenous applications.

The clinical data collected include the following demographic and clinical characteristics: age, sex, body mass index (BMI), age at first episode, current episode duration, past substance abuse, past alcohol use disorder, and family history of psychiatric disorders.

### Ketamine Treatment

Participants received 0.5 mg/kg ketamine via infusion over 40 min. Of 26 subjects, 18 received only one ketamine treatment, six received two ketamine infusions, one received three ketamine administrations, and one received a total of five ketamine infusions. Multiple ketamine administrations were separated by periods of 4 to 42 weeks. Here, we present only findings from the first ketamine treatment.

### Clinical Response

Clinicians assessed depression symptoms using MADRS (Montgomery and Asberg, 1979) at three time points: at baseline, 24 h after treatment, and 7 days after treatment. In addition, at each time point, participants filled out Beck Depression Inventory (BDI-II) (Beck et al., 1996). We quantified treatment outcome after 24 h as percent MADRS total score change from baseline. We categorized participants as responders if they displayed an improvement of 50% or more of their baseline MADRS score after 24 h, and as non-responders otherwise.

### Statistical Analysis

First, we tested normality for each quantified variable with Shapiro–Wilk test, assuming that *p*-values < 0.05, uncorrected for multiple comparisons, reflect significant deviations from normal distribution. Second, we tested the effect of ketamine on MADRS total score with a repeated-measures analysis of variance (ANOVA). We analyzed the correlation between MADRS total score change 24 h and 7 days after ketamine treatment and age, age of first episode, and BMI. We ran one-tailed tests for the correlation between BMI and percent MADRS total score improvement, based on previous reports. Next, we classified all subjects as responders versus non-responders according to a MADRS total score improvement of 50% or more after 24 h. Finally, for each BDI-II item we compared score changes from baseline to 24 h and 7 days after treatment in responders to score changes in non-responders. To this end, we used Welch tests and Mann-Whitney tests (one-tailed, expecting larger differences in responders, uncorrected for multiple testing). We supplemented statistical analyses with measures of effect size. For all inferential and descriptive statistics, we reported exact values rounded to the third decimal.

We carried out all statistical analyses in R (R Core Team. R Foundation for Statistical Computing, Vienna, Austria)^1^. In addition to core R functionalities, we used package “ez” for repeated measures analysis of variance, package “effsize” to calculate effect sizes, and packages “lattice,” “latticeExtra,” “gridExtra,” and “ggplot2” to plot figures.

## Results

### Study Sample

Table 1 presents a summary of demographic and clinical characteristics. We tested 26 participants (13 female) with a mean age of 48.5 years (range: 24–73). Mean age of first episode was 27.923 years (range: 6–64). Mean MADRS total score at baseline was 26.846 (range: 11–36). Twenty-two participants fulfilled diagnostic criteria for recurrent major depressive disorder (F33). Three subjects met diagnostic criteria for bipolar disorder with a current depressive episode (F31.3), and one participant was diagnosed with another depressive episode (F32.8).

**TABLE 1:** Clinical characteristics.

Sample characteristic	Descriptive statistics
Sample size	26^1^
Age	48.5 ± 2.818 (24–73)^2^
Age of first episode	27.923 ± 2.69 (6–64)^2^
Current episode duration	3.078 ± 1.012 (0.038–23)^2^
Body mass index	25.488 ± 0.917 (19.152–40.152)^2^
MADRS score at baseline	26.846 ± 1.194 (11–36)^2^
Female subjects	13^1^
Subjects with Major Depressive Disorder	23^1^
Subjects with Bipolar Disorder	3^1^
Family history of psychiatric disorders	17^1^
History of alcohol abuse	2^1^

*^1^Indicates number of participants (count); ^2^Indicates mean ± standard error of the mean with range in parentheses: (minimum–maximum).*

### Data Distribution

Shapiro-Wilk tests did not indicate significant deviations from normality for MADRS and BDI-II total scores, MADRS and BDI-II total score changes from baseline to 24 h after treatment, age, and age of first episode (*p*-values > 0.05, uncorrected for multiple comparisons, Supplementary Tables S1–S3). In contrast, current episode duration, MADRS and BDI-II percent total score changes from baseline to 7 days after treatment, MADRS and BDI-II item scores at each of the three timepoints, and BMI deviated significantly from normality (*p*-values < 0.05, uncorrected for multiple comparisons, Supplementary Tables S1–S3). Therefore, in the following, we supplemented the results of parametric tests with non-parametric procedures, to exclude statistical artifacts, caused by skewed distributions, in parametric tests.

### Symptom Improvements After Ketamine Treatment as Measured With MADRS

Mean MADRS total score decreased from 26.8 at baseline (range: 11–36, *n* = 26) to 17.5 at 24 h (range: 0–35, *n* = 26) and 18.6 at 7 days (range: 0–34, *n* = 25) after ketamine treatment. These changes were statistically significant (Supplementary Table S4 and Supplementary Figure S1). *Post hoc t*-tests and Wilcoxon signed-rank tests showed significant decreases from baseline to 24 h and 7 days after treatment (Supplementary Tables S5, S6). We found no significant difference between MADRS total scores 24 h and 7 days after ketamine treatment, indicating a sustained improvement over a week (Supplementary Tables S5, S6).

We found considerable interindividual variability in the response to ketamine treatment. MADRS total score changes varied from a 3.0% increase to a complete remission (reduction of 100%) after 7 days. Nine of the 26 participants fulfilled response criteria after 24 h, corresponding to a fraction of 34.6%. Importantly, response to ketamine was stable over time: eight of the nine responders still fulfilled response criteria after 7 days (88.9%). Only one out of nine responders at 24 h post-treatment did not meet response criteria after 7 days (11.1%), and only 1 out of 16 non-responders after 24 h was categorized as a responder 7 days after ketamine treatment (6.3%). MADRS total score percent change from baseline to 24 h after treatment closely corresponded to MADRS total score percent change from baseline to 7 days after treatment (Pearson’s *r* = 0.832, *p* < 0.001, two-tailed; Spearman’s rho = 0.862, *p* < 0.001, two-tailed; *n* = 25, one non-responder at 24 h could not be tested 7 days after ketamine treatment because she left the treatment center).

Correlation analyses indicated that higher BMI corresponded to higher MADRS percent total score improvement after 24 h and 7 days (Supplementary Tables S7, S8).

### MADRS Item Profile of Symptom Improvements After Ketamine Treatment

Parametric explorative analyses revealed a rather unspecific effect of ketamine on MADRS scores at the item level (Supplementary Tables S9, S10). Item scores decreased significantly for all MADRS items after 24 h, except for item 5 (“Reduced Appetite”) (*p* = 0.096, two-tailed, uncorrected for multiple comparison).

### Comparisons Between Responders and Non-responders

Responders and non-responders did not differ significantly in their total MADRS and BDI-II scores at baseline (Supplementary Table S11). Comparisons at the item level indicated higher scores in non-responders in item 13 (“Indecisiveness,” *p* = 0.26, two-tailed, uncorrected for multiple comparisons) and item 19 (“Concentration,” *p* = 0.49, two-tailed, uncorrected for multiple comparisons). Non-parametric comparisons corroborated the difference in item 13 and suggested a trend for item 19 (Supplementary Table S11).

**FIGURE 1:**
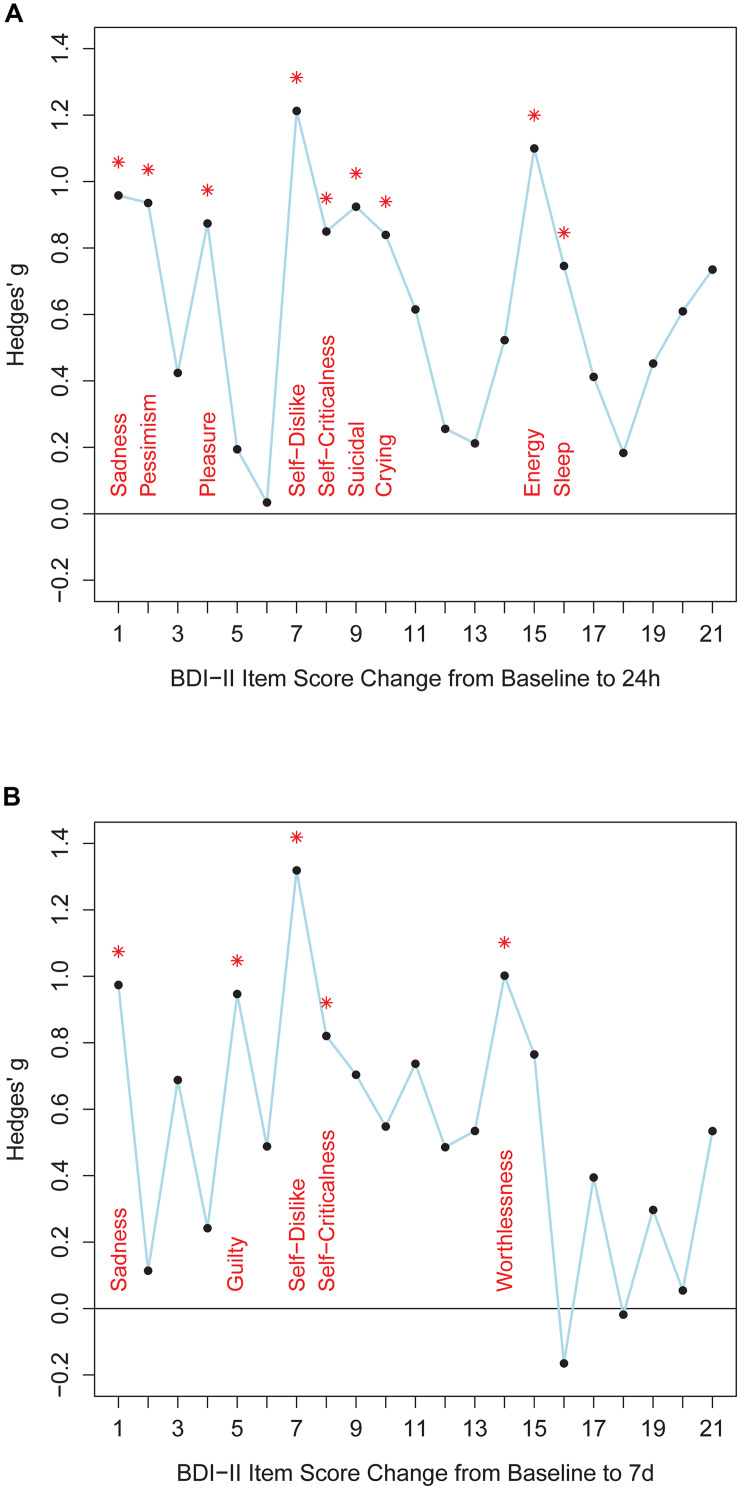
The figure shows effect sizes for comparisons between responders and non-responders. Panel **(A)** shows BDI-II item score changes from baseline to 24 h after ketamine treatment. Panel **(B)** shows BDI-II item score changes from baseline to 7 days after ketamine treatment. *BDI-II items 1-21* are shown on the *x*-axis. *Hedges’ g* effect size measures are plotted along the *y*-axis (calculated with R package “effsize”). *Positive values* indicate better item score improvements in responders than in non-responders. *Asterisks* indicate significant differences between item score improvement of responders and non-responders (Welch tests, *p* < 0.05, one-tailed, uncorrected for multiple comparisons, test statistics are provided in Supplementary Table S12).

Twenty-four hours after ketamine treatment responders showed better improvements than non-responders on the following BDI-II items: sadness, pessimistic thoughts, loss of pleasure, self-dislike, self-criticalness, suicidal thoughts, increased crying, loss of energy, and changes in sleep pattern (Figure 1 and Supplementary Table S12). Non-parametric tests confirmed this item response profile (Supplementary Table S13). Seven days after ketamine treatment responders still displayed better improvement than non-responders in the items sadness, guilty, self-dislike, self-criticalness, and worthlessness (Figure 1 and Supplementary Table S12). Non-parametric tests confirmed these results and showed a better symptom improvement in responders in three further items: failure, agitation, and loss of energy (Supplementary Table S13). Thus, overall, responders improved significantly more than non-responders both 24 h and 7 days after ketamine treatment on the following BDI-II items: item 1 (“Sadness”), item 7 (“Self-Dislike”), and item 8 (“Self-Criticalness”) (Figure 1; *p*-values < 0.05, one-tailed, uncorrected for multiple comparisons, Supplementary Table S12). Mean score change from baseline to 24 h after ketamine treatment for non-responders and responders was, respectively, −0.47 and −1.333 for item 1 (“Sadness”), −0.176 and −1.111 for item 7 (“Self-Dislike”), −0.294 and −1 for item 8 (“Self-Criticalness”) (negative values indicating a score decrease). Mean score change from baseline to 7 days after ketamine treatment for non-responders and responders was, respectively, −0.188 and −1.111 for item 1 (“Sadness”), 0.125 and −0.667 for item 7 (“Self-Dislike”), −0.125 and −0.889 for item 8 (“Self-Criticalness”) (negative values indicating a score decrease).

## Discussion

In an open-label study, 26 participants with treatment-resistant depression received 0.5 mg/kg ketamine via infusion. Mean MADRS total score was 26.846 at baseline (range: 11–36, *n* = 26) and dropped to 17.538 (range: 0–35, *n* = 26) 24 h after treatment, and 18.64 (range: 0–34, *n* = 25) 7 days after treatment. After 24 h nine of the 26 participants fulfilled response criteria. Of these, eight remained responders after 7 days. Only one of 16 non-responders, according to MADRS total score improvement after 24 h, fulfilled response criteria after 7 days. Higher MADRS percent total score change from baseline to 24 h after ketamine treatment corresponded to higher BMI. Responders improved significantly more than non-responders 24 h and 7 days after ketamine treatment in the BDI-II items “Sadness” (item 1), “Self-Dislike” (item 7), and “Self-Criticalness” (item 8).

Our results corroborate the study by Ballard et al. (2018). They combined several studies in major depression and bipolar depression and examined unique symptom clusters regarding treatment response to ketamine. Ketamine’s strongest effects were found on the clusters “negative cognition,” “depressed mood,” “amotivation,” “anhedonia,” and “suicidal thoughts,” whereas effects on the symptom clusters “tension,” “impaired sleep,” and “reduced appetite” were less pronounced. The symptom cluster “negative cognition” included eight BDI items: “Thoughts of Failure,” “Guilt,” “Feelings of Punishment,” “Self-Criticalness,” “Increased Crying,” “Worthlessness,” “Reduced Sexual Interest,” and “Disappointment in Self.” The first seven of those items are also present in BDI-II, which we used in our study. We observed a better improvement in responders than non-responders in the items “Self-Criticalness,” “Self-Dislike,” “Pessimistic Thoughts,” “Increased Crying,” and “Worthlessness.” Overall, our findings largely support an improvement in this symptom cluster. Moreover, we found higher symptom improvement in the BDI-II item “Sadness,” in line with the effects of ketamine on the symptom cluster “depressed mood” reported by Ballard et al. (2018) We also found a better improvement in responders than non-responders in the BDI-II item “Suicidal Thoughts,” consistent with the reported effect on the symptom cluster “suicidal thoughts” (Ballard et al., 2018). Overall, our findings are consistent with the reported effects of ketamine on symptom clusters “negative cognition,” “depressed mood,” and “suicidal thoughts” (Ballard et al., 2018).

Because our study period was longer (7 days versus 3 days), we added to Ballard et al. (2018) report that most improvements in negative self-schema were not only rapid but also sustained over one week. In contrast to a previous meta-analysis on ketamine’s effects on the BDI item “Suicidal Thoughts” (Wilkinson et al., 2018), we found a significant improvement on this item in BDI-II, after 24 h but not after 7 days.

In an integrative hypothesis of depression, Price and Duman (2020) suggested that impaired neuroplasticity in depression may be the neural substrate of rigid negative biases, including negative appraisals of the self, which is a fundamental component of the negative triad of depression. In his cognitive theory of depression, (Beck, 1979) emphasized the central role of negative self-schemata, a negative view of others, and negative expectations about the future that significantly impair emotional wellbeing and functioning. Taken together, our data suggest that ketamine might positively influence dysfunctional self-schemata. Ketamine may have the potential to enhance cognitive-behavioral psychotherapy in treatment-resistant depression (Hasler, 2019).

Consistent with previous meta-analyses (Han et al., 2016; Kishimoto et al., 2016), we showed rapid and robust symptom improvements following a single dose of ketamine as assessed with MADRS. Our response rate of 34.6% was slightly lower than previously reported, possibly because we defined treatment resistance as non-response to at least three different treatments. We found a high correlation of *r* = 0.832 (*p* < 0.001) between symptom reductions after 24 h and after 7 days, suggesting that antidepressant effects were sustained and not identical with ketamine’s acute alterations of self-perception and consciousness (Stocker et al., 2019). This finding indicates that clinical response after 24 h is an important predictor of clinical response after 7 days.

High BMI was associated with high MADRS total score improvement after ketamine, which is consistent with previous evidence that high BMI is one of the most replicable predictors of the response to intravenous ketamine treatment (Rong et al., 2018).

Several limitations of our study merit comments. First, the lack of a placebo control condition is a major limitation. Whether the observed decrease in depression scores after treatment reflects the therapeutic action of ketamine, or unspecific placebo effects, remains unclear (Kirsch, 2019). In addition, the lack of a control group does not allow controlling for the effects of regression to the mean on the average decrease of MADRS total scores we observed. A second limitation of our study is multiple testing without *p*-value correction. This increases the risk for false positive findings and warrants caution when interpreting results. On the other hand, consecutive conservative correction for 21 comparisons over three time points would have considerably reduced statistical power and increased the risk for type II “false negative” errors. Moreover, we found an item-response profile after 24 h that remained stable after 7 days. Responders improved more than non-responders on BDI-II items “Sadness,” “Self-Dislike,” “Self-Criticalness,” and “Energy loss” 24 h and 7 days after ketamine treatment. Furthermore, the BDI-II item profile we observed could be partly driven by different item properties, such as different reliabilities, different item score variances, and differences in item score correlations with baseline and post-measurements. The use of percent score change from baseline, difference scores, and the concept of responders and non-responders have received critical evaluation (Vickers, 2001; Snapinn and Jiang, 2007; Clifton and Clifton, 2019). Therefore, results obtained with these measures should be regarded with caution. The significant violations of normality assumption we observed for several variables represent another limitation. Skewed distributions might produce false positive findings in parametric tests. To rule this out we supplemented analyses with extensive non-parametric testing that corroborated most of the results from parametric procedures.

Our results are based on the effects of ketamine that is a racemic mixture containing equal amounts of the enantiomers esketamine and arketamine. A dose of 0.25 mg/kg esketamine had a similar short-term antidepressant effects and a similar safety profile to 0.5 mg/kg ketamine, suggesting that esketamine is the active enantiomer (Correia-Melo et al., 2020). However, there is preliminary evidence that arketamine might provide a more prolonged antidepressant response than esketamine with less side effects, particularly with no dissociative and hemodynamic effects. Since the clinical trials on esketamine and arketamine did not include BDI as an outcome measure, more research is needed to discuss the role of the two enantiomers regarding their differential therapeutic effects on negative self-schema in depression.

In summary, our findings suggest that ketamine may act therapeutically in treatment resistant depression by improving self-related cognitive biases. This is a potentially important finding since negative self-schemata are at the core of cognitive theories of depression (Beck, 1979) and associated with impaired neuroplasticity in depression (Price and Duman, 2020). Independent replications in larger samples using placebo-controlled study designs are needed to corroborate our findings.

## Data Availability Statement

The raw data supporting the conclusions of this article will be made available by the authors, without undue reservation, to any qualified researcher.

## Ethics Statement

The studies involving human participants were reviewed and approved by Kantonale Ethikkommission Bern. The patients/participants provided their written informed consent to participate in this study.

## Author Contributions

SS, GH, and GS administered ketamine and acquired data. YM, GH, and SS analyzed the data and prepared all figures. GH and YM wrote the main manuscript. All authors reviewed and contributed to the manuscript.

## Conflict of Interest

GH received research support, consulting fees, and speaker honoraria from Lundbeck, Sunovion, Servier, Janssen, and Vifor. The remaining authors declare that the research was conducted in the absence of any commercial or financial relationships that could be construed as a potential conflict of interest.

## Footnotes

1 https://www.R-project.org/
