# Supplementary material for: Sustained Improvement of Negative Self-Schema After a Single Ketamine Infusion: An Open-Label Study
Source: Front Neurosci. 2020 Jul 1;14:687. doi: 10.3389/fnins.2020.00687 (PMC7341975; doi:10.3389/fnins.2020.00687)
Supplement: Supplementary file 1 [file Data_Sheet_1.pdf]

Supplementary Material

1.1 Supplementary Tables

Supplementary Table S1. Normality of MADRS Scores

MADRS Score	Shapiro-Wilk p-value at baseline (n = 26)	Shapiro-Wilk p-value after 24 hours (n = 26)	Shapiro-Wilk p-value after 7 days (n = 25)
MADRS Total Score	0.361456224	0.416445618	0.483175219
MADRS Item 1	0.00829085	0.003398134	0.002261153
MADRS Item 2	0.107847064	0.00092545	0.006289953
MADRS Item 3	0.029853488	0.014107216	0.009225003
MADRS Item 4	0.008991902	0.000897127	0.000340752
MADRS Item 5	0.001700153	0.003614123	0.004858464
MADRS Item 6	0.009306187	0.123961709	0.121773891
MADRS Item 7	0.014133003	0.005225729	0.04843569
MADRS Item 8	0.039404623	0.050471354	0.110978265
MADRS Item 9	0.008536171	0.003946468	0.040674982
MADRS Item 10	0.005499376	0.000124231	0.00034034

p-values indicate p-values from Shapiro-Wilk normality tests, uncorrected for multiple comparisons.

Supplementary Table S2. Normality of BDI-II Scores

BDI-II Score	Shapiro-Wilk p-value at baseline (n = 26)	Shapiro-Wilk p-value after 24 hours (n = 26)	Shapiro-Wilk p-value after 7 days (n = 25)
BDI-II Total Score	0.822848121	0.641312152	0.750617411
BDI-II Item 1	0.006010062	3.79061E-05	8.38448E-05
BDI-II Item 2	0.00677334	0.000398814	0.001452703
BDI-II Item 3	0.007221622	0.00058198	0.002259114
BDI-II Item 4	0.001262247	0.003766479	0.004853642
BDI-II Item 5	0.001263289	0.000144879	0.001250001
BDI-II Item 6	2.88613E-05	4.84071E-06	1.65008E-05
BDI-II Item 7	0.002754234	5.91956E-05	0.001633654
BDI-II Item 8	0.003596203	0.000217968	0.001820368
BDI-II Item 9	9.95113E-05	3.26555E-07	5.82835E-05
BDI-II Item 10	0.002326457	0.000552298	0.000158608
BDI-II Item 11	7.98754E-05	8.03181E-05	9.18596E-05
BDI-II Item 12	0.001188244	0.003324969	0.003198312
BDI-II Item 13	0.000217968	0.001871873	0.007554778
BDI-II Item 14	0.001300392	0.000121415	0.000112841
BDI-II Item 15	5.47482E-05	0.000230982	0.00052738
BDI-II Item 16	0.000571834	0.000257195	0.000703729
BDI-II Item 17	0.002247647	3.97121E-05	0.000436089
BDI-II Item 18	0.001990731	6.80973E-05	0.002158112
BDI-II Item 19	0.000933677	0.004771299	6.73329E-05
BDI-II Item 20	0.001637394	0.001829548	0.007477488
BDI-II Item 21	0.000530875	0.000607583	0.002792592

p-values indicate p-values from Shapiro-Wilk normality tests, uncorrected for multiple comparisons.

Supplementary Table S3. Normality of Demographic and Other Clinical Variables

Variable	Sample Size (n)	Shapiro-Wilk p-value
Age	26	0.521844526
Age of First Episode	26	0.383939425
Duration of Current Episode in Years	26	1.03053E-07
BMI	26	0.024997803
MADRS Percent Score Change Baseline – 24 h	26	0.827374724
MADRS Percent Score Change Baseline – 7 d	25	0.01309749
BDI-II Percent Score Change Baseline – 24 h	26	0.20083504
BDI-II Percent Score Change Baseline – 7 d	25	0.01718624

p-values indicate p-values from Shapiro-Wilk normality tests, uncorrected for multiple comparisons.

Supplementary Table S4. Repeated Measures Analysis of Variance

Effect	F	df_1	df_2	p-value
Timepoint	30.541	2	48	< 0.001

Results of a repeated measures analysis of variance (ANOVA), as calculated with R package "ez". ***F*** indicates F statistic, df_1 and df_2 indicate the corresponding degrees of freedom, ***p-value*** indicates the corresponding p-value. One person was omitted from analysis, resulting in n = 25.

Supplementary Table S5. Comparisons Between MADRS Total Scores With Within-Subject t-Tests

Comparison	Df	t-value	p-value	Effect size
Baseline – 24 hours post	25	6.357	< 0.001	1.119
Baseline – 7 days post	24	5.864	< 0.001	0.916
24 hours post – 7 days post	24	-1.381	0.180	-0.138

Within-subject t-tests calculated in R. ***Df***, degrees of freedom; ***t-value***, t-statistic; ***p-value***, two-tailed, uncorrected for multiple comparisons; ***Effect size***, Hedges' *g* as calculated in R with package "effsize" (pooled variance).

Supplementary Table S6. Nonparametric Comparisons Between MADRS Total Scores With Wilcoxon Signed-Rank Tests

Comparison	n	V	p-value
Baseline – 24 hours post	26	270	< 0.001
Baseline – 7 days post	25	297.5	< 0.001
24 hours post – 7 days post	25	70.5	0.119

Within-subject t-tests calculated in R. ***n***, sample size; ***V***, V-statistic; ***p-value***, two-tailed, uncorrected for multiple comparisons.

Supplementary Table S7. Correlations of MADRS Total Score Change from Baseline to 24 Hours after Treatment with BMI, Age, and Age of First Episode

Correlation with	Pearson's r	p-value	Spearman's rho	p-value
BMI	0.4112078	0.018 (one-tailed)	0.526604	0.003 (one-tailed)
Age	-0.04152815	0.840 (two-tailed)	-0.07874016	0.702 (two-tailed)
Age of First Episode	-0.2590882	0.201 (two-tailed)	-0.2208904	0.278 (two-tailed)

Supplementary Table S8. Correlations of MADRS Total Score Change from Baseline to 7 Days after Treatment with BMI, Age, and Age of First Episode

Correlation with	Pearson's r	p-value	Spearman's rho	p-value
BMI	0.2213547	0.144 (one-tailed)	0.3459023	0.045 (one-tailed)
Age	0.09988653	0.635 (two-tailed)	0.1135708	0.589 (two-tailed)
Age of First Episode	-0.1330993	0.526 (two-tailed)	-0.1675333	0.423 (two-tailed)

Supplementary Table S9. MADRS Item Score Changes from Baseline to 24 Hours and Seven Days After Treatment, Analysed With Within-Subject t-Tests

Item	Label	t-value Baseline-24h	p-value Baseline-24h	ES Baseline-24h	t-value Baseline-7d	p-value Baseline-7d	ES Baseline-7d	t-value 24h-7d	p-value 24h-7d	ES 24h-7d
1	Apparent Sadness	5.333333333	1.57704E-05	0.896867963	5.881176353	4.56678E-06	0.987746626	0.810884854	0.42540045	0.106947719
2	Reported Sadness	4.697010726	8.1714E-05	0.816788293	3.078832937	0.00514191	0.546594213	-1.564724487	0.130738895	-0.254037292
3	Inattention	4.255512714	0.000256462	1.017360666	2.722178615	0.011885728	0.594863308	-2.19089023	0.038406945	-0.345063828
4	Reduced Sleep	2.609312292	0.015100854	0.40444637	1.692829478	0.103431222	0.192972546	-1.189270634	0.245966746	-0.212943144
5	Reduced Appetite	1.72928616	0.096089049	0.272448131	2.652259934	0.013948313	0.498234269	1.809068067	0.082985261	0.194551239
6	Concentration	2.559960558	0.016895709	0.568259204	3.464101615	0.002013253	0.613405566	0.170664037	0.865918566	0.030635662
7	Lassitude	4.35483871	0.000198358	0.849648517	5.765745074	6.07773E-06	0.964456087	0.617758401	0.542552569	0.091815681
8	Inability to Feel	3.441047556	0.002046209	0.804421288	3.160719504	0.004223168	0.666839927	-0.595183621	0.557291647	-0.100958622
9	Pessimistic Thoughts	4.395245365	0.000178656	0.923344061	2.4931561	0.01995573	0.641069972	-1.673664464	0.107182234	-0.230817412
10	Suicidal Thoughts	3.067859955	0.005126745	0.639013399	1.673664464	0.107182234	0.35244102	-1.661494321	0.109623188	-0.296188509

Item, MADRS Item 1-10, ***t-values***, ***p-values***, and ***ES*** indicate t-statistics, the corresponding two-tailed p-values uncorrected for multiple comparisons, and Hedges' *g* effect size-measures (as calculated with R package "effsize") for the following comparisons: ***Baseline-24h***, Baseline – 24 hours after ketamine treatment (n = 26); ***Baseline-7d***, Baseline – seven days after ketamine treatment (n = 25); ***24h-7d***, 24 hours after ketamine treatment – seven days after ketamine treatment (n = 25). Positive t-values indicate an improvement over time (item score decrease), whereas negative t-values indicate a worsening of symptoms over time (item score increase).

Supplementary Table S10. MADRS Item Score Changes from Baseline to 24 Hours and Seven Days After Treatment, Analysed With Wilcoxon Signed-Rank Tests

Item	Label	V Baseline-24h	p-value Baseline-24h	V Baseline-7d	p-value Baseline-7d	V 24h-7d	p-value 24h-7d
1	Apparent Sadness	153	0.000265793	210	6.18729E-05	55	0.499037985
2	Reported Sadness	160	0.001018953	106.5	0.007679922	12.5	0.132949834
3	Inattention	174	0.001294607	116	0.011656675	13.5	0.041734552
4	Reduced Sleep	52	0.01258148	57	0.154067558	8	0.347323533
5	Reduced Appetite	36	0.116914044	52	0.012942565	36	0.095335425
6	Concentration	112	0.022051845	98	0.004031605	35.5	0.85468235
7	Lassitude	171.5	0.001750841	171	0.000139563	27	0.624422604
8	Inability to Feel	165	0.004736776	108	0.006237716	25	0.495131061
9	Pessimistic Thoughts	148.5	0.000597814	151.5	0.022270979	24	0.114716598
10	Suicidal Thoughts	81	0.012252171	76.5	0.123371171	13	0.137256455

Item, MADRS Item 1-10, ***V*** and ***p-values*** indicate test statistics and the corresponding two-tailed p-values, uncorrected for multiple comparisons. Comparisons annotated as in Supplementary Table S9.

Supplementary Table S11. Itemwise Parametric and Nonparametric Comparisons Between Responders and Non-Responders for BDI-II Item Scores at Baseline

Item	Label	t-value	p-value (t-test)	ES	W	p-value (U-test)
	Total Score	1.4673	0.1589	0.5586163	99	0.2352
1	Sadness	0.213276467	0.833555975	0.082960444	81.5	0.79725421
2	Pessimism	1.357712461	0.191410855	0.525248017	102.5	0.146918165
3	Failure	0.833350288	0.415233207	0.318179826	88.5	0.515405605
4	Pleasure	1.379693838	0.184730245	0.534574026	102	0.154457643
5	Guilty	0.030141717	0.976357264	0.012500114	76.5	1
6	Punishment	0.948100995	0.355826967	0.368210372	95	0.294490387
7	Self-Dislike	-0.723439345	0.482351836	-0.316414166	64.5	0.50977481
8	Self-Criticalness	0.061410505	0.951740724	0.024113701	77.5	0.977539922
9	Suicidal	-0.482624139	0.639079084	-0.228654317	74.5	0.928808679
10	Crying	0.742816831	0.4709709	0.325256414	89	0.502023879
11	Agitation	0.424662376	0.677432547	0.178616401	83.5	0.686249238
12	Interest	1.665414131	0.112719513	0.637481483	104.5	0.113687272
13	Indecisiveness	2.443022657	0.026195663	0.974804527	116	0.025327324
14	Worthlessness	0.903269867	0.37908346	0.356892919	91	0.421730411
15	Energy	1.011021403	0.329951005	0.435542329	95	0.26166485
16	Sleep	0.617561518	0.54531089	0.24624961	85	0.641737288
17	Irritability	0.056298953	0.955602135	0.019886239	78.5	0.931835499
18	Appetite	0.688955785	0.501717483	0.287663918	87	0.558722589
19	Concentration	2.134760439	0.049220671	0.871825062	109	0.055979492
20	Fatigue	1.134147929	0.275366784	0.475975379	96.5	0.27239345
21	Sex	1.422077647	0.1729604	0.559118155	101	0.176984786

Item, indicates BDI-II Items 1-21, and corresponding ***Label***. ***t-value*** and ***p-value (t-test)*** show t-statistics (Welch test) and the corresponding two-tailed p-values, uncorrected for multiple comparisons, between responders and non-responders. Positive ***t-values*** indicate higher BDI-II item score in non-responders than in responders. ***ES***, indicates Hedges' *g* effect size calculated with R package "effsize". ***W*** and ***p-value (Mann-Whitney U-test)*** show test statistics and the corresponding two-tailed p-values, uncorrected for multiple comparisons. For all comparisons n = 26.

Supplementary Table S12. Itemwise Parametric Comparisons Between Responders and Non-Responders for BDI-II Item Score Changes from Baseline to 24 Hours and to Seven Days After Treatment

Item	Label	t-value 24h-Baseline	p-value 24h-Baseline	ES 24h-Baseline	t-value 7d-Baseline	p-value 7d-Baseline	ES 7d-Baseline
1	Sadness	2.408474427	0.013992736	0.958508474712366	2.404862769	0.014140063	0.974358974358974
2	Pessimism	1.990481583	0.03612505	0.935896659515293	0.251196028	0.402973195	0.114065158359917
3	Failure	1.088397536	0.145581472	0.424427698761828	1.501633098	0.079914021	0.687849700051954
4	Pleasure	2.28411899	0.017202413	0.87422125496356	0.543462304	0.298171642	0.24200741290581
5	Guilty	0.55116384	0.293472419	0.194198474187617	1.967697888	0.038279046	0.947090675146967
6	Punishment	0.08202096	0.467880937	0.034377503715465	1.040406034	0.160286931	0.488294726645643
7	Self-Dislike	2.704076162	0.009522676	1.21316457227731	2.629773529	0.013188177	1.31885395432783
8	Self-Criticalness	2.051706755	0.029146218	0.849815532880371	1.885818971	0.040624983	0.820588414734958
9	Suicidal	1.904747545	0.042711496	0.924719848798792	1.425683359	0.092594623	0.703783164258268
10	Crying	1.936691755	0.037283547	0.839792781631035	1.252889835	0.116025119	0.548117809652362
11	Agitation	1.351130632	0.101124249	0.615414725445091	1.664356663	0.060223388	0.736983228715563
12	Interest	0.664402818	0.257407465	0.256158999904719	1.203888718	0.122734279	0.485676766320976
13	Indecisiveness	0.504814259	0.310699097	0.212150260192497	1.460964251	0.079236228	0.534668255169292
14	Worthlessness	1.220185637	0.121596331	0.522822804388563	2.359615814	0.01648871	1.00188442880164
15	Energy	2.553214606	0.011813087	1.09991047997451	1.760914715	0.050505588	0.765041340189613
16	Sleep	1.964052253	0.032225906	0.746051766497669	-0.383482494	0.646405875	-0.164794459768527
17	Irritability	1.069929573	0.149343969	0.412131843133374	0.984062725	0.16946242	0.394614521441374
18	Appetite	0.415900196	0.342234902	0.183372876995646	-0.048876533	0.519259644	-0.0181183336713624
19	Concentration	1.101123596	0.144008189	0.452376266341183	0.689730495	0.250912561	0.296949737441444
20	Fatigue	1.383503706	0.095249906	0.609995035644729	0.141990459	0.444289011	0.0546561390581524
21	Sex	1.626544216	0.065012206	0.735128613628637	1.158362623	0.13516288	0.534197256291467

Item, indicates BDI-II Items 1-21, and corresponding ***Label***. ***t-values*** and ***p-values*** show t-statistics and the corresponding one-tailed p-values, uncorrected for multiple comparisons, between responders and non-responders; ***24h-Baseline***, indicates itemwise comparisons between responders and non-responders for Item score difference 24 hours after ketamine treatment - baseline (n = 26); ***7d-Baseline***, indicates itemwise comparisons between responders and non-responders for Item score difference 7 days after ketamine treatment - baseline (n = 25). ***ES***, indicates Hedges' *g* effect size calculated with R package "effsize". Positive t-values indicate higher BDI-II item score improvement in responders than in non-responders.

Supplementary Table S13. Itemwise Nonparametric Comparisons Between Responders and Non-Responders for BDI-II Item Score Changes from Baseline to 24 Hours and to Seven Days After Treatment

Item	Label	W-value 24h-Baseline	p-value 24h-Baseline	W-value 7d-Baseline	p-value 7d-Baseline
1	Sadness	116.5	0.01198435	111	0.010460452
2	Pessimism	117.5	0.008799811	75.5	0.426641311
3	Failure	97.5	0.108654046	99.5	0.045056869
4	Pleasure	113.5	0.014851341	84	0.232257842
5	Guilty	89	0.235234876	104.5	0.016940459
6	Punishment	77.5	0.486828579	86.5	0.144683314
7	Self-Dislike	123.5	0.002390904	109	0.002886506
8	Self-Criticalness	111	0.019920867	102	0.034589246
9	Suicidal	107	0.030806907	91	0.06683842
10	Crying	105	0.026324295	84	0.222907949
11	Agitation	95.5	0.124485311	97	0.031859127
12	Interest	86	0.287973089	91.5	0.113436744
13	Indecisiveness	84.5	0.319037326	96	0.068021518
14	Worthlessness	97	0.117216133	108	0.01098337
15	Energy	114	0.007796593	98	0.048013419
16	Sleep	108.5	0.025373469	74	0.46147124
17	Irritability	99.5	0.089505052	91	0.109093242
18	Appetite	84.5	0.308915567	73.5	0.474330264
19	Concentration	95	0.124580787	79	0.331922744
20	Fatigue	97	0.099107608	76	0.408491143
21	Sex	101	0.055227729	86	0.184633363

Item, indicates BDI-II Items 1-21, and corresponding ***Label***. ***W-values*** and ***p-values*** show test statistics and the corresponding one-tailed p-values, uncorrected for multiple comparisons between responders and non-responders with Mann-Whitney tests; ***24h-Baseline***, indicates itemwise comparisons between responders and non-responders for item score difference 24 hours after ketamine treatment - baseline (n = 26); ***7d-Baseline***, indicates itemwise comparisons between responders and non-responders for item score difference 7 days after ketamine treatment - baseline (n = 25).

1.2 Supplementary Figures

Supplementary Figure S1

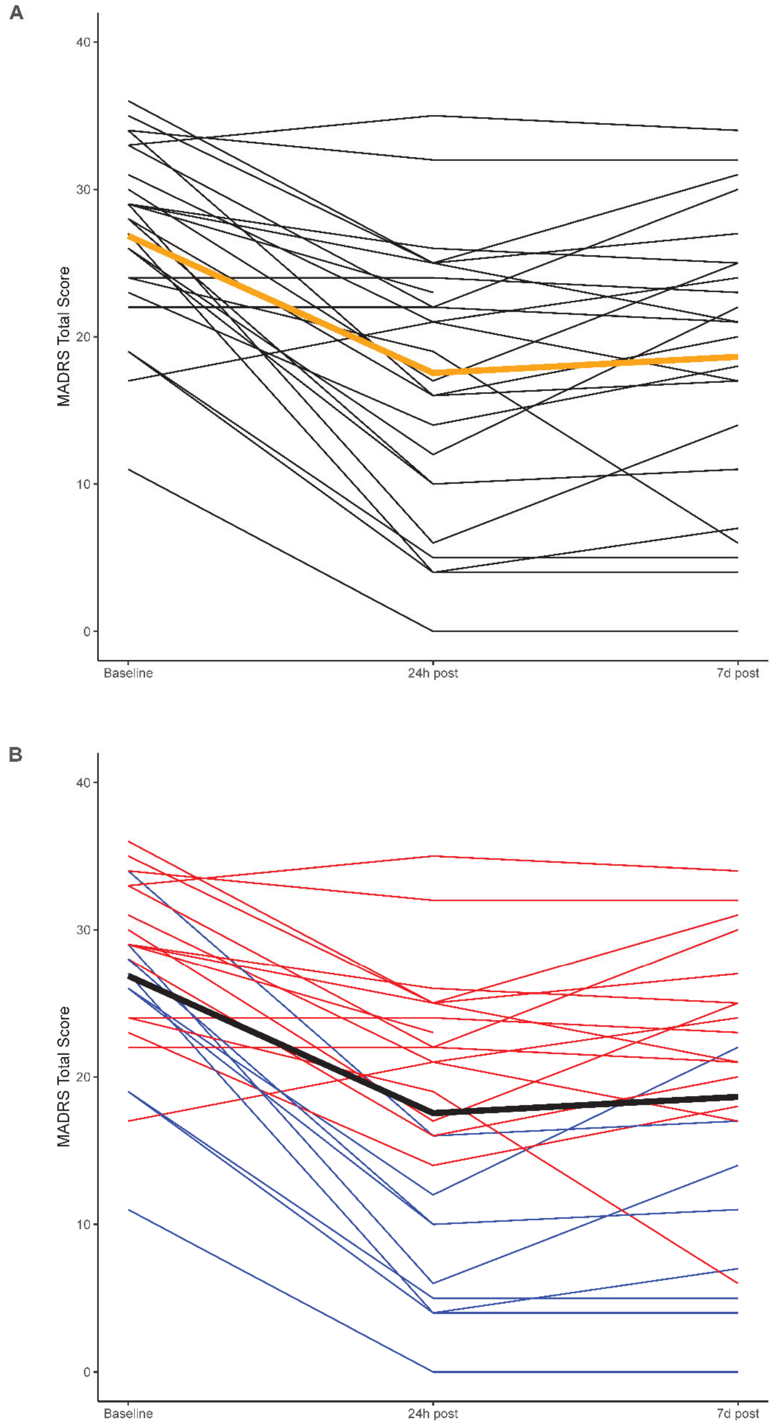

The figure shows changes in MADRS total score from baseline (***Baseline***) to 24 hours (***24 h post***) and seven days (***7d post***) after ketamine treatment at the individual level. **Panel A: *Black lines*** correspond do individual participants, the ***orange line*** shows changes in the average MADRS total score. One participant was tested at baseline and after 24 hours, but not after seven days. ***MADRS*** total scores are shown on the y-axis. **Panel B: *Red lines*** represent non-responders, ***blue lines*** represent responders, the ***black line*** shows changes in the average MADRS total score.

Supplementary Figure S2

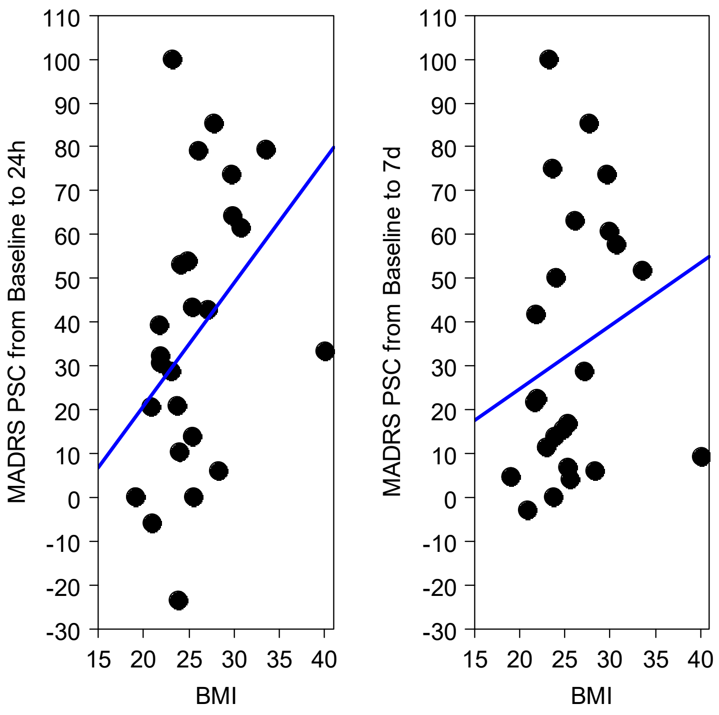

Scatterplots show the relationship between MADRS Percent Total Score Change from Baseline to 24 hours (***MADRS PSC from Baseline to 24h***, left panel) and from Baseline to seven days (***MADRS PSC from Baseline to 7d***, right panel) after ketamine treatment. ***Black dots*** depict individual participants, ***blue lines*** indicate linear regression estimates, as calculated with R package "lattice". For corresponding correlation tests, see Supplementary Tables S7 and S8.
